# Bone metabolism during strict head-down tilt bed rest and exposure to elevated levels of ambient CO_2_

**DOI:** 10.1038/s41526-022-00245-0

**Published:** 2022-12-16

**Authors:** Emily R. McGrath, Petra Frings-Meuthen, Jean Sibonga, Martina Heer, Gilles R. Clement, Edwin Mulder, Scott M. Smith, Sara R. Zwart

**Affiliations:** 1 Cornell University, Ithaca, NY USA; 2 German Aerospace Center, Cologne, Germany; 3 NASA Johnson Space Center, Houston, TX USA; 4 IU International University of Applied Sciences and University of Bonn, Bad Reichenhall, Germany; 5 KBR, Houston, TX USA; 6 University of Texas Medical Branch, Galveston, TX USA

**Keywords:** Physiology, Epidemiology

## Abstract

Astronauts on the International Space Station are exposed to levels of atmospheric carbon dioxide (CO_2_) above typical terrestrial levels. We explored the possibility that increased levels of ambient CO_2_ further stimulate bone resorption during bed rest. We report here data from 2 ground-based spaceflight analog studies in which 12 male and 7 female subjects were placed in a strict 6° head-down tilt (HDT) position for either 30 days at 0.5% ambient CO_2_ or 60 days with nominal environmental exposure to CO_2_. Bone mineral density (BMD) and bone mineral content (BMC) were determined using dual-energy X-ray absorptiometry (DXA). Blood and urine were collected before and after HDT for biochemical analysis. No change was detected in either BMD or BMC, as expected given the study duration. Bone resorption markers increased after bed rest as expected; however, elevated CO_2_ had no additive effect. Elevated CO_2_ did not affect concentrations of minerals in serum and urine. Serum parathyroid hormone and 1,25-dihydroxyvitamin D were both reduced after bed rest, likely secondary to calcium efflux from bone. In summary, exposure to 0.5% CO_2_ for 30 days did not exacerbate the typical bone resorption response observed after HDT bed rest. Furthermore, results from these strict HDT studies were similar to data from previous bed rest studies, confirming that strict 30–60 days of HDT can be used to evaluate changes in bone metabolism. This is valuable in the continuing effort to develop and refine efficacious countermeasure protocols to mitigate bone loss during spaceflight in low-Earth orbit and beyond.

## Introduction

Space is a hostile environment that brings many health risks for astronauts. The loss of bone mineral and muscle tissue are particularly well-documented outcomes of spaceflight^1–4^. Long-duration space missions on Skylab, Mir, and the International Space Station (ISS) have all resulted in some degree of bone loss^1,5–9^. Kidney calcification and renal stone formation are potential byproducts because calcium is released from bone when the body is unloaded in a microgravity environment^1,5,6,8,10^. To ensure the success of future human space exploration missions, it is important to understand the factors that might induce detrimental changes in the mass and the structure of bone so countermeasures can be developed to mitigate these changes.

One of the factors that could contribute to spaceflight-induced bone loss is exposure to elevated atmospheric carbon dioxide (CO_2_). Airflow is limited in spacecrafts and resources to scrub CO_2_ from the atmosphere are constrained, which is conducive to higher ambient CO_2_ levels^11^. In fact, on the ISS, CO_2_ levels are often about 0.5% (5000 ppm)^11^, over 10 times higher than ambient outdoor terrestrial levels^12^. Exposure to increased levels of CO_2_ can result in many health issues, including cognition deficits, headaches, and detrimental effects on bone health^11,12^.

Bed rest is a common analog used to assess human physiology responses to the microgravity environment of spaceflight. The bone hyperresorption induced by bed rest is well-documented to reflect hyperresorption induced by actual spaceflight, although at a lesser magnitude^1,7,8,13,14^. Subjects are placed in a 6° head-down tilt (HDT) position during bed rest, which reduces the typical mechanical forces on the bone, leading to bone resorption as the bone adapts to decreased weight and pressure^15^. Because bone exists in a dynamic equilibrium between formation and resorption, under reduced mechanical loading, the bone adjusts to the decreased need for strength by increasing resorption, which then outpaces formation, leading to bone loss^16^. Although bed rest is not a quantitative replica of spaceflight conditions, it produces the same qualitative effects such as bone loss^1,17–19^, decreased calcium absorption^20^ and increased urinary and fecal excretion of calcium^13,14,20,21^, leading to a negative calcium balance^7,9,20^. During many previous bed rest studies, subjects were allowed to prop their head up using a pillow; however to maintain the 6° angle on the head during strict HDT, no pillows were used. This was initiated to increase headward fluid pressure to more closely mimic the physiological effects of spaceflight.

A state of acidosis in the body induces several factors that affect bone metabolism, and these could exacerbate spaceflight or bed-rest induced bone loss. It is hypothesized that CO_2_ levels between 0.7–1.0%^22^ and diets with high potential renal acid load (PRAL) may trigger metabolic acidosis^23,24^. Bone acts as a buffer during metabolic acidosis by stimulating osteoclasts to release calcium carbonate to negate the acidosis^25^, resulting in hypercalciuria^11,26^ and a negative calcium balance^27^. CO_2_ levels greater than 0.7–1.0%^27^ prompt cyclic periods of respiratory and metabolic acidosis^22,28^ mirrored by fluctuations in the levels of circulating calcium and phosphorus as they are released from bone^29^. Contrary to metabolic acidosis, respiratory acidosis triggers renal absorption of bicarbonate to buffer the increased pCO_2_^22^, and conflicting results have been reported regarding the effects on calcium regulation^27^. Some studies report an increase in urinary calcium^28,30^, whereas others report calcium retention^11^ or minimal to no change at all^31^ in response to respiratory acidosis. The precise level of CO_2_ known to trigger metabolic or respiratory acidosis in humans has never been clarified as it largely depends on individual metabolic responses to the exposure.

To our knowledge no studies have examined how CO_2_ levels affect bone loss during bed rest. Thus, we sought to determine how bone responds to 0.5% CO_2_ during strict HDT bed rest. Because the average ambient concentration of CO_2_ on the ISS is 0.5%^12^, the results of this study can help interpret the qualitative physiological changes in astronauts on the ISS. Guinea pig exposure to 0.5% CO_2_ for 8 weeks has resulted in kidney calcification and elevated plasma calcium levels, thus indicating 0.5% CO_2_ has a measurable physiological impact^32^. While it is unlikely metabolic or respiratory acidosis will be observed at such low levels of exposure, kidney calcification and calcium efflux from bone are both direct health risks to astronauts. Elevated CO_2_ exposure will continue to be a risk as space exploration missions extend beyond low-Earth orbit, so it is important to evaluate how CO_2_ impacts the body to determine an acceptable exposure level.

## Results

### Densitometry

The body mass of the elevated CO_2_ group and the control group was the same after strict bed rest as it was before bed rest (Table 1). As expected, 30–60 days of bed rest induced only minor changes in bone densitometry findings in both groups of subjects (Table 1).

**Table 1:** Body mass and bone densitometry in strict head-down tilt bed rest with or without CO_2_ exposure.

	Control Pre (*n* = 8)	Control Post 60 Days (*n* = 8)	CO_2_ Pre (*n* = 11)	CO_2_ Post 30 Days (*n* = 11)
Femoral Neck BMD (g/cm^2^)	1.144 ± 0.147	1.129 ± 0.144	1.028 ± 0.147	1.025 ± 0.139
Whole Body BMC (g)	3230 ± 497	3195 ± 471^a^	2694 ± 480	2702 ± 472
Whole Body BMD (g/cm^2^)	1.321 ± 0.082	1.328 ± 0.086	1.251 ± 0.102	1.256 ± 0.109
Body mass (kg)	80.1 ± 12.7	80.2 ± 13.2	70.9 ± 8.6	69.2 ± 7.9

Data are means ± SD.

^a^Statistically different from before bed rest, as determined by two-way repeated measures ANOVA analysis, *p* < 0.05.

### Biochemistry

**Fig. 1:**
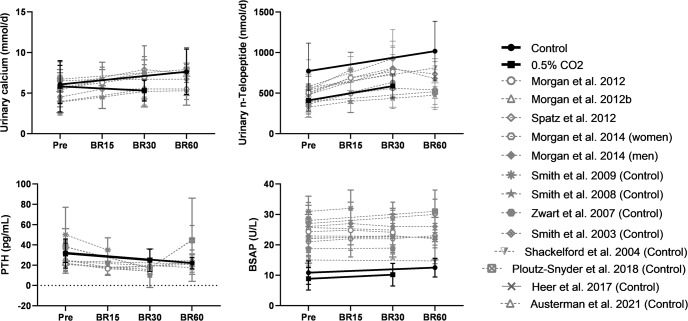
Bone biochemistry. Urinary calcium, n-telopeptide, parathyroid hormone (PTH), and bone-specific alkaline phosphatase (BSAP) before (Pre), and during bed rest (BR15, BR30, or BR60) comparing several bed rest studies of similar duration. Data are means ± SD. The control data from this study are the solid black line and filled circle (AGBRESA Control) and the subjects exposed to 0.5% CO_2_ during bed rest are the solid line with filled squares. Comparable bed rest studies are in gray with dashed lines^9,13,14,16,18,21,33–35,47,65,66^.

Bone resorption, as assessed from levels of excreted collagen crosslinks, increased after 30 and 60 days of strict HDT bed rest, but was not exacerbated by exposure to elevated levels of CO_2_ (Table 2). Collagen crosslinks N-telopeptide (NTX) excretion was greater in the control group before and after bed rest than the excretion levels in the CO_2_ group (expressed as ug/g, *P* < 0.05, or nmol/d, post-hoc *P* < 0.01). Deoxypyridinoline (DPD) excretion following bed rest was greater in the control groups compared to the CO_2_ group (*P* < 0.05); however, this interaction effect was only observed when the data were normalized to creatinine (Table 2). Levels of crosslink excretion in these 2 strict HDT studies were similar to values reported for earlier bed rest studies^7,13,14,21,33–36^ (Fig. 1).

**Table 2:** Bone markers during strict head-down tilt bed rest with or without CO_2_ exposure.

	Control Pre (*n* = 8)	Control Post 60 Days (*n* = 8)	CO_2_ Pre (*n* = 11)	CO_2_ Post 30 Days (*n* = 11)	Study Effect (CO_2_ vs Control)	BR Effect (Pre vs BR)	Interaction Effect (StudyxBR)
NTX (ug/g Cr)	693 ± 326^b^	1160 ± 451^a,b^	474 ± 114	789 ± 237^a^	<0.05	<0.0001	ns
NTX (nmol/d)	772 ± 344^b^	1017 ± 368^a,b^	410 ± 128	586 ± 147^a^	<0.01	<0.001	ns
CTX (ug/g Cr)	1120 ± 670	2230 ± 803^a^	1170 ± 385	1790 ± 470^a^	ns	<0.0001	ns
CTX (nmol/d)	2620 ± 1500	3990 ± 1200^a^	2190 ± 1040	2950 ± 1210^a^	ns	<0.001	ns
PYD (ug/g Cr)	105 ± 44	247 ± 80^a^	136 ± 33	224 ± 78^a^	ns	<0.0001	ns
PYD (nmol/d)	539 ± 8328	890 ± 163^a^	483 ± 119	719 ± 281^a^	ns	<0.001	ns
DPD (ug/g Cr)	22.6 ± 11.8	55.9 ± 15.8^a,b^	25.9 ± 7.5	41.2 ± 14.6^a^	ns	<0.0001	<0.05
DPD (nmol/d)	129 ± 99^b^	211 ± 36^a,b^	96 ± 28	139 ± 58^a^	<0.05	0.001	ns
PTH (pg/mL)	32.2 ± 9.6	22.1 ± 5.6^a^	31.5 ± 14.3	25.0 ± 10.9^a^	ns	0.001	ns
Osteocalcin (ng/mL)	12.4 ± 3.27	14.5 ± 2.36^a,b^	12.6 ± 2.30	11.8 ± 1.85	ns	ns	<0.01
P1NP (ug/L)	68.5 ± 26.5	94.1 ± 31.9^a^	56.2 ± 13.3	63.6 ± 14.5	<0.05	<0.0001	<0.01
BSAP (ug/L)	10.8 ± 3.82	12.5 ± 3.06	8.84 ± 3.67	10.2 ± 3.75	ns	ns	ns

Data are means ± SD.

*ns* no statistical significance.

^a^Significantly different from pre BR, as determined by two-way repeated measures ANOVA, *p* < 0.05.

^b^Significantly different from CO_2_, as determined by two-way repeated measures ANOVA, *p* < 0.05.

Bone-specific alkaline phosphatase (BSAP), a marker of bone formation, tended to increase after bed rest but did not reach statistical significance (post-hoc *P* = 0.09, Table 2). However, after bed rest, serum type 1 procollagen (P1NP) concentration, another indication of bone formation, increased 44 ± 35% above pre-bed rest levels in the control group (interaction *P* < 0.01), and increased 14 ± 15% above pre-bed rest levels in the elevated CO_2_ group (Table 2). Concentrations of serum osteocalcin in the control group were 19 ± 14% higher on the last day of bed rest than they were before bed rest, but were unchanged after bed rest in the elevated CO_2_ group (interaction *P* < 0.01) (Table 2). Concentrations of circulating parathyroid hormone (PTH) decreased after bed rest by 27 ± 26% and 19 ± 20% in the control and elevated CO_2_ groups, respectively (post-hoc *P* < 0.001). Similarly, concentrations of 1,25 (OH)_2_ vitamin D in both groups of subjects were less after bed rest than before bed rest (−29 ± 11% and −34 ± 18% in the control and in the elevated CO_2_ groups, respectively). In the control group, 25(OH) vitamin D levels were 13 ± 10% higher after bed rest than before bed rest, but no differences were detected in 25(OH) vitamin D levels in the elevated CO_2_ group. Bed rest-induced changes in serum vitamin K were also different in each group of subjects (interaction *P* < 0.001): the control group had a significant increase in serum vitamin K after bed rest (104 ± 85%) as compared to pre-bed rest values, whereas no changes were detected in the elevated CO_2_ group (Table 3). Although concentrations of magnesium in the serum were unaffected by bed rest or treatment, levels of magnesium in the urine were lower after bed rest than before, especially in the control group where urine magnesium was 40 ± 13% lower than values before bed rest (interaction *P* < 0.01) (Table 3). Before bed rest, the control subjects had higher levels of magnesium in their urine than the elevated CO_2_ subjects (interaction *P* < 0.01). Levels of serum calcium were higher (post-hoc *P* < 0.001) after bed rest than before bed rest but values were unaffected by CO_2_ exposure. Excretion of urinary calcium tended to increase in the control group and decrease in the elevated CO_2_ group after bed rest (interaction *P* = 0.058). For both groups of subjects, levels of serum phosphorus were higher (post-hoc *P* < 0.001) after bed rest than before bed rest, whereas urine phosphate was lower after bed rest but decreased to a greater extent in the control group (interaction *P* < 0.01). The serum concentrations of potassium and sodium were unaffected by bed rest and CO_2_ exposure, but sodium levels in urine were lower in both groups after bed rest (post-hoc *P* < 0.001), and potassium levels in the urine were lower in the control group after bed rest (interaction *P* < 0.05). Other chemistry results are shown in Table 2.

**Table 3:** Biochemical markers of bone and calcium metabolism during strict head-down tilt bed rest with or without CO_2_ exposure.

	Control Pre (*n* = 8)	Control Post 60 Days (*n* = 8)	CO_2_ Pre (*n* = 11)	CO_2_ Post 30 Days (*n* = 11)	Study Effect (CO_2_ vs Control)	BR Effect (Pre vs BR)	Interaction Effect (StudyxBR)
Vitamin Status							
K1 (ng/L)	519 ± 220	1010 ± 483 ^a,b^	350 ± 363	133 ± 61	<0.001	ns	<0.001
25(OH) Vitamin D (µg/L)	28.6 ± 6.1	32.1 ± 6.7 ^a^	33.1 ± 4.3	31.3 ± 5.5	ns	ns	<0.01
1,25(OH)_2_ Vitamin D (pg/mL)	50.8 ± 9.9	35.6 ± 6.3^a^	52.9 ± 17.3	33.4 ± 8.2^a^	ns	<0.0001	ns
Urinalysis							
pH	5.13 ± 0.35^b^	5.00 ± 0.00^b^	5.73 ± 0.47	5.36 ± 0.51	<0.001	ns	ns
Sodium (mmol/d)	113 ± 20.3	43.6 ± 10.3^a^	112 ± 24.4	60.8 ± 10.8^a^	ns	<0.0001	ns
Potassium (mmol/d)	78.8 ± 23.3	53.2 ± 15.7^a^	66.1 ± 12.4	57.8 ± 12.2	ns	<0.0001	<0.05
Uric Acid (mmol/d)	4.58 ± 2.23	3.87 ± 0.73	3.74 ± 0.94	3.66 ± 0.79	ns	ns	ns
Urea (mmol/d)	409 ± 92.2^b^	345 ± 107^a^	269 ± 56.0	325 ± 91.1^a^	<0.05	ns	<0.001
Serum Mineral Status							
Zinc (µg/L)	721 ± 87	883 ± 159^a^	717 ± 124	855 ± 133^a^	ns	<0.0001	ns
Phosphorus (mmol/L)	1.26 ± 0.10	1.39 ± 0.16^a^	1.28 ± 0.13	1.40 ± 0.09^a^	ns	<0.001	ns
Magnesium (mmol/L)	0.82 ± 0.04	0.81 ± 0.05	0.85 ± 0.05	0.84 ± 0.03	ns	ns	ns
Calcium (mmol/L)	2.29 ± 0.09	2.35 ± 0.07^a^	2.34 ± 0.07	2.39 ± 0.06^a^	ns	<0.001	ns
Urine Mineral Status							
Magnesium (mmol/d)	8.87 ± 3.93^b^	5.10 ± 2.04^a^	4.53 ± 1.55	3.75 ± 1.14	ns	<0.0001	<0.01
Calcium (mmol/d)	6.07 ± 2.34	7.63 ± 2.79	5.83 ± 3.16	5.31 ± 1.28	ns	ns	ns
Phosphate (mmol/d)	41.7 ± 12.0	14.6 ± 6.1^a,b^	33.0 ± 13.1	20.0 ± 6.1^a^	ns	<0.0001	<0.01
Zinc (μmol/d)	12.7 ± 5.2^b^	17.0 ± 7.4^a,b^	5.5 ± 3.5	7.6 ± 3.7^a^	<0.001	<0.05	ns
Serum Chemistry							
Alkaline Phosphatase (U/L)	64.2 ± 12.8	80.0 ± 14.1^a,b^	61.9 ± 15.4	64.7 ± 14.4	ns	<0.01	<0.05
Sodium (mmol/L)	140 ± 1.0	141 ± 2.2	139 ± 1.4	137 ± 1.3 ^a,b^	<0.001	ns	<0.01
Potassium (mmol/L)	4.37 ± 0.41	4.38 ± 0.69	4.26 ± 0.29	4.40 ± 0.52	ns	ns	ns
Chloride (mmol/L)	105 ± 2.1	100 ± 0.5	103 ± 1.9	101 ± 2.3^a^	<0.01	<0.05	<0.05
Uric Acid (mg/dL)	5.87 ± 1.11^b^	5.39 ± 1.13^a,b^	4.59 ± 1.20	4.45 ± 1.03^a^	<0.05	<0.01	ns
Blood Urea Nitrogen (mg/dL)	11.5 ± 1.7^b^	12.1 ± 1.7^b^	21.3 ± 5.3	24.3 ± 4.3^a^	<0.0001	<0.01	<0.05

Data are means ± SD.

*ns* no statistical significance.

^a^Significantly different from pre BR, as determined by two-way repeated measures ANOVA test, *p* < 0.05.

^b^Significantly different from CO_2_, as determined by two-way repeated measures ANOVA test, *p* < 0.05.

Blood pH increased slightly, but significantly, in the elevated CO_2_ group after bed rest (interaction *P* < 0.01), rising 0.3 ± 1.4%. The capillary whole blood bicarbonate concentration increased (interaction *P* < 0.01) after bed rest (11 ± 6%) in the elevated CO_2_ group, whereas no change was observed in the control group. The pCO_2_ increased after bed rest (post-hoc *P* < 0.01) in both groups of subjects (Table 4).

**Table 4:** Capillary blood during strict head-down tilt bed rest with or without CO_2_ exposure. Data are means ± SD.

	Control Pre (*n* = 8)	Control Post 60 Days (*n* = 8)	CO_2_ Pre (*n* = 11)	CO_2_ Post 30 Days (*n* = 11)	Study Effect (CO_2_ vs Control)	BR Effect (Pre vs BR)	Interaction Effect (StudyxBR)
pH	7.43 ± 0.03	7.41 ± 0.02^b^	7.42 ± 0.02	7.44 ± 0.02^a^	ns	ns	<0.01
pO_2_ (mmHg)	76.5 ± 4.3	82.8 ± 13.0	75.8 ± 6.3	75.3 ± 7.5	ns	ns	ns
pCO_2_ (mmHg)	39.5 ± 5.0	41.3 ± 3.4^a^	41.0 ± 3.2	43.4 ± 4.2^a^	ns	<0.01	ns
HCO_3_ (mmol/L)	25.3 ± 2.3	25.7 ± 1.0^b^	26.3 ± 2.3	29.1 ± 2.7^a^	<0.05	<0.001	<0.01
Anion gap (mmol/L)	10.2 ± 1.5	7.4 ± 1.7^ab^	10.3 ± 1.3	10.9 ± 1.8	<0.001	ns	<0.05

*ns* no statistical significance.

^a^Significantly different from pre BR, as determined by two-way repeated measures ANOVA test, *p* < 0.05.

^b^Significantly different from CO_2_, as determined by two-way repeated measures ANOVA test, *p* < 0.05.

### Diet

Dietary intake data are reported in Table 5. Vitamin K intake was similar for both groups of subjects before bed rest, and a small but significant decline was detected in the elevated CO_2_ group (interaction *P* < 0.001).

**Table 5:** Average dietary intake before, during, and after strict head-down tilt bed rest with or without CO_2_ exposure.

	Control Pre (*n* = 8)	Control Bed Rest (*n* = 8)	Control Post 60 Days (*n* = 8)	CO_2_ Pre (*n* = 11)	CO_2_ Bed Rest (*n* = 11)	CO_2_ Post 30 Days (*n* = 11)	Requirements^a^
Total Energy (kcal/d)	2740 ± 352	2430 ± 334	2810 ± 362	2530 ± 339	2040 ± 300	2450 ± 373	
Protein (g/kg BW/d)	1.20 ± 0.01	1.17 ± 0.04	1.19 ± 0.03	1.20 ± 0.03	1.19 ± 0.04	1.20 ± 0.06	1.2
Protein (% of total energy)	14.1 ± 1.5	15.6 ± 1.7	13.7 ± 1.7	14.2 ± 1.0	17.4 ± 1.5	14.5 ± 1.3	
Total fat (% of total energy)	32.2 ± 0.3	31.6 ± 0.7	32.3 ± 0.9	33.1 ± 0.9	32.5 ± 1.0	33.0 ± 0.9	30–35
Carbohydrates (% of total energy)	50.9 ± 1.4	50.0 ± 1.6	51.1 ± 1.5	52.7 ± 1.3	50.1 ± 1.8	52.5 ± 1.5	50–60 %TEE^b^
Fluid (ml/d)	4000 ± 580	4030 ± 497	4640 ± 552	3580 ± 366	3560 ± 373	4080 ± 633	
Fluid (ml/kg BW/d)	50.5 ± 1.3	51.0 ± 3.4	58.8 ± 3.2	50.6 ± 2.4	50.5 ± 3.2	57.8 ± 7.5	50
Calcium (mg/d)	1080 ± 9	1080 ± 24	1090 ± 11	1050 ± 59	1060 ± 35	1090 ± 39	1000–12,000
Iron (mg/d)	19.0 ± 1.5	19.5 ± 1.2	20.0 ± 1.3	17.8 ± 2.6	17.8 ± 4.0	17.8 ± 3.9	10 (M), 18 (F)
Potassium (mg/d)	4030 ± 344	3720 ± 336	4070 ± 390	4340 ± 480	3660 ± 459	3880 ± 416	3500–5000
Magnesium (mg/d)	455 ± 28	448 ± 41	477 ± 42	457 ± 64	408 ± 59	436 ± 73	300
Sodium (mg/d)	2960 ± 11	2910 ± 69	2930 ± 82	2950 ± 75	2930 ± 115	2940 ± 77	2500–3000
Phosphorus (mg/d)	1580 ± 152	1540 ± 196	1590 ± 201	1490 ± 191	1380 ± 191	1450 ± 219	700–1700
Zinc (mg/d)	13.6 ± 0.6	13.5 ± 0.9	14.2 ± 0.8	13.7 ± 2.3	12.7 ± 2.9	13.6 ± 2.7	12–15
Vitamin D (µg/d)	29.4 ± 0.4	28.6 ± 0.5	29.1 ± 0.3	27.9 ± 1.6	27.8 ± 1.8	28.6 ± 2.2	25
Vitamin K (µg/d)	193 ± 15	211 ± 26	202 ± 11	234 ± 125	210 ± 136	233 ± 154	80
Absolute PRAL (g/d)	46.6 ± 7.1	45.8 ± 7.9	46.4 ± 7.9	41.6 ± 4.9	41.0 ± 5.1	41.5 ± 5.1	
Body Weight PRAL (g/d/Kg BW)	0.59 ± 0.01	0.58 ± 0.02	0.58 ± 0.02	0.59 ± 0.01	0.58 ± 0.01	0.59 ± 0.01	

Data are means ± SD.

^a^Requirements defined by VaPER study in coordination with standards developed by NASA and ESA.

^b^% total energy expenditure.

## Discussion

Exposure to 0.5% CO_2_ for 30 days did not exacerbate markers predicting bone loss during strict HDT bed rest. However, this study did corroborate previous physiological effects of prolonged bed rest without strict HDT, including elevated markers of bone resorption^8,9,13,15,16^, increased urinary calcium excretion^13,14,35^, and decreased concentrations of serum PTH^9,13,16,19,20,26,33^ and 1,25 vitamin D^1,16,20^ that are often observed in bed rest studies of similar duration. In the present study, urinary calcium tended to increase after bed rest in the control group (*P* = 0.058), but did not reach statistical significance.

No measurable change was detected in the density or content of bone mineral in either the elevated CO_2_ group or the control group. This was expected, however, because it typically takes 90 days or more of bed rest for measurable changes to occur in bone mineral content (BMC) and bone mineral density (BMD) using DXA. The lack of change is consistent with data from other bed rest studies of similar durations^9,14,21^.

Whereas imaging techniques cannot quantify the small degree of bone loss that occurs in short-term studies such as the one reported here, collagen crosslinks provide biochemical evidence of bone resorption that ultimately lead to BMD or BMC changes, and the crosslinks respond within first days of bed rest^37^. An interaction effect was detected in the crosslink DPD when normalized to ug/g creatinine in that DPD increased more in the control group after bed rest. The interaction effect was likely due to subject variation because creatinine may yield abnormal results from a myriad of nonspecific factors which could interfere with the creatinine estimate up to 15%. These factors include simple day-to-day variations, emotional stress, or menstrual cycles, all of which may have impacted the subjects. A bed rest effect but no interaction effect was detected when DPD was expressed in nmol/d. NTX is often considered a more reliable marker of bone resorption, and the percent increases in NTX during the control (37.1 ± 31.0%) and the CO_2_ (55.0 ± 67.5%) studies were within the typical 50-65% range of NTX increase reported for previous bed rest studies^13,14,16,35,38^ (Fig. 1).

Collagen crosslinks provide specific insight into bone resorption, whereas urinary calcium is a non-specific marker affected by many variables. Bed rest and elevated CO_2_ exposure had no effect on calcium excretion; however, modest increases in calcium levels were detected in the serum of both groups of subjects. PTH and active vitamin D levels both decreased as well, likely secondary to the calcium release from bone^13,19^. The level of 25(OH) vitamin D, a marker of vitamin D stores in the body, was significantly higher after bed rest than before bed rest in the control group, whereas the level of 25(OH) vitamin D decreased modestly in the CO_2_ group. Although not attributable to the CO_2_ exposure, these findings reinforce the impact of bed rest and decreased mechanical loading on calcium regulation and bone metabolism.

Although subjects who do not exercise during bed rest typically have no change or a slight decrease in the levels of bone formation markers while their levels of bone resorption markers and osteoclast activity rises^39^, our data show mixed results^15^. PTH decreased in both groups, as typically observed during bed rest; however, P1NP increased and BSAP tended to increase in both groups (*P* = 0.09), which is more consistent with bed rest studies where subjects are exercising^40^ or when formation and resorption are still coupled. It is possible that the CO_2_ exposure and bed rest did not lead to the complete uncoupling of bone formation and resorption, such that the rapid increase of bone resorption triggered a modest increase in formation, somewhat repressed in the CO_2_ group. However, other bed rest studies do not report increases of bone formation markers after bed rest, so this result will need to be interpreted further in other studies.

D. Bemben et al.^41^ examined bone marker responses utilizing the CO_2_ dataset, but without the control data. D. Bemben et al. similarly found that bone turnover increased as indicated by the significant increase of P1NP and serum calcium. Our data indicates that while P1NP demonstrates an interactive effect when compared to control data, the serum calcium change becomes nonsignificant. Similarly, D. Bemben et al. found that PTH significantly decreased and bone alkaline phosphatase (bone ALP) increased significantly only in women. Similar to the results of D. Bemben et al., we observed a significant interactive effect with bone ALP. However unlike D. Bemben et al., we found the PTH becomes nonsignificant when compared to the control. D. Bemben found that the bone ALP was only significant in women, which may explain our interaction effect since the number of women in the CO_2_ group outnumbered those in the control. While osteocalcin and 25(OH) vitamin D were determined nonsignificant by D. Bemben et al., we observed an interaction effect in both when compared to control data. Although we did not observe significant changes in BMD, D. Bemben et al. found total hip aBMD significantly decreased and was significantly greater in females.

As CO_2_ levels rise above terrestrial levels (e.g., ~0.04%), the body eventually enters a state of metabolic acidosis diagnosed through arterial levels of pH and bicarbonate that provoke changes in bone metabolism. The anion gap may also be calculated, as a high anion gap may signify metabolic acidosis^42^. A statistically significant interaction effect with group and bed rest was found for both blood pH and bicarbonate that were measured in capillary blood pH, confirming previously published data of arterialized pH^43^. In comparison studies, both capillary pH and bicarbonate have been found to have a strong and significant positive correlation with arterial values^44^. Thus, the capillary measures are a good indication of arterial dissolved gas trends. Blood pH increased in the CO_2_ group and decreased in the control group. Blood bicarbonate increased in both groups. The calculated anion gap remained constant in the CO_2_ subjects but decreased in the control subjects, which provides evidence that exposure to 0.5% CO_2_ for 30 days did not increase metabolic acidosis. The small decrease in anion gap could perhaps be explained by a change in albumin status^42^.

If metabolic acidosis were present, we could expect hypercalciuria and changes in levels of serum sodium and potassium. In chronic metabolic acidosis, serum phosphate would also rise^25^. No hypercalciuria was observed beyond the effects of bed rest. Urine potassium decreased only in the control group, and urine phosphate decreased the most in the control group, reinforcing that the 30-day exposure to 0.5% CO_2_ did not trigger a metabolic acidosis response. Respiratory acidosis also likely did not occur because PTH typically increases during respiratory acidosis and our subjects had decreased levels of PTH after bed rest^45^.

The lack of day-to-day biochemistry sampling is a limitation of this study. Any acute biochemical changes would not be evident in the current study because measures were only taken on the last day of bed rest.

The small differences in dietary intake could potentially explain the different effects of bed rest and CO_2_ exposure on serum vitamin K and osteocalcin. Vitamin K is the coenzyme essential for producing osteocalcin, so it is possible the increase in osteocalcin in the control group was due to differences in vitamin K intake. This is supported in literature which demonstrates that vitamin K intake has a significant impact on bone turnover in spaceflight^46^. It is also possible that the differing durations of HDT between the groups had an effect on osteocalcin.

Our data show that exposure to 0.5% CO_2_ does not exacerbate markers predicting bone loss during 30 days of bed rest. A buffering or acidosis response from bone has been observed with higher levels of CO_2_ exposure, particularly above 1.0%^11,28^, and it is possible that CO_2_ levels of 0.5% are too low to induce this effect. The threshold level for CO_2_-induced effects on bone loss has still to be determined for both spaceflight and terrestrial conditions.

Studies of bone mineral and muscle tissue loss during bed rest have been conducted for almost 75 years, and the design and implementation of these studies has varied with location, collaborating studies, duration, degree (if any) of HDT, and many other factors. Previous studies have assessed bone loss during 17 weeks of horizontal bed rest^17,47,48^, because the investigators of these studies believed that disuse, and not the fluid shifts induced by HDT, is what induces bone loss during bed rest. In the 2000s, an international group of researchers established standard bed rest protocols that employ 6° HDT in an attempt to mimic the fluid shifts induced by spaceflight^49^. When trying to develop a bed rest model for astronaut ocular pathologies, investigators believed that standard 6° HDT bed rest was limiting the fluid pressures in the head because subjects were using a pillow to prop their head up and they leant on an elbow while eating. The studies reported here followed what has been coined “strict HDT,” which places subjects at a 6° angle without the use of a pillow under the head. The strict adherence preserves the angle of the head, actually changing the estimated arterial blood pressure on the head from 100 mmHg at 0° to 105 mmHg at 6°^50^. This pressure change may contribute to the resulting optic disc edema observed in some bed rest subjects^51^. The data reported here match those from earlier horizontal bed rest studies (Fig. 1), even with the elevated CO_2_ group only exposed to HDT for 30 days and the control group exposed for 60 days. This suggests that strict HDT bed rest for 30–60 days can also be used to study unloading-induced changes in bone metabolism. This is a point of significance due to reported difficulties in finding and utilizing good models for simulating microgravity^52^.

This research combined biochemical, densitometry, and dietary intake data to study bone metabolism during strict HDT and elevated CO_2_ exposure. No evidence was detected for loss of bone mass that would affect the integrity of the bone due to exposure to 0.50% CO_2_ atmosphere alone, although longer-duration studies might reveal otherwise. This strict HDT study design induced similar levels of bone biochemistry responses as earlier bed rest studies without HDT. Additionally, serum 1,25(OH)_2_ vitamin D and PTH were reduced after bed rest in both groups of subjects, suggesting that calcium efflux from bone was indeed having a physiological impact, but there was no effect specific to CO_2_ exposure alone that would lead to bone mineral loss. Future research on bone metabolism at other CO_2_ levels is needed to better establish a risk ceiling, especially as astronauts explore beyond low-Earth orbit in vehicles that will no doubt have CO_2_ levels higher than terrestrial exposures.

## Methods

Data were obtained from the NASA Human Research Program Standard Measures Cross-Cutting Project from 2 bed rest studies: the vision impairment/intracranial pressure (VIIP) and Psychological:envihab Research Study (VaPER), a joint study between NASA and the German Aerospace Center (DLR); and the Artificial Gravity Bed Rest Study (AGBRESA), a joint study between NASA, European Space Agency (ESA), and the DLR. The studies were conducted at the DLR:envihab facility, which was designed to evaluate the physiological effects of bed rest, including bone loss. Although these overarching studies included many individual experiments, the data reported here were obtained from the NASA Standard Measures Project. Other results from these bed rest studies have been published^53^; however, none of the data reported herein have been previously published. NASA’s and DLR’s Institutional Review Board approved the experimental protocol and all subjects gave their written informed consent prior to participating in the study.

### Bed rest studies

The VaPER study tested the physiological effects of a 30-day exposure to strict 6° HDT, and 0.5% ambient CO_2_ levels. VaPER consisted of a one-campaign, one-armed bed rest study without a cross-over design and lacking an ambulatory and a nominal environmental CO_2_ exposed control group. 11 subjects (6 male and 5 female) completed the study. Subjects underwent a 14-day baseline data collection phase (BDC) before bed rest, 30 days of sustained 6° HDT for all activities, followed by re-ambulation and recovery for 14 days. Dietary intake was standardized and controlled in accordance with the International Bed Rest Standards^54^. The study was performed from October 12 to December 4, 2017.

Inclusion criteria included subjects who were physically and mentally healthy, aged between 24–55 y with a BMI of 19–30 kg/m^2^. Subjects were required to be non-smokers for at least 6 months, and successfully complete a modified Air Force Class 3 physical. Exclusion criterial included any drug, medication, or alcohol abuse, vegetarian, have migraines, claustrophobia, eye disorders, gastro-esophageal reflux, chronic back complaints, bone fractures in either tibia or radii, muscle or joint disorder, diabetes, or history of chronic bowel disease.

The AGBRESA bed rest study tested the physiological effects of a 60-day exposure to strict 6° HDT. Although some subjects were exposed to continuous or intermittent artificial gravity treatment via centrifugation, this report only includes the 8 control subjects (2 women, 6 men) who received no artificial gravity exposure. AGBRESA consisted of 2 bed-rest campaigns, the first carried out between March 25 (first day of BDC 15 days before bed rest) and June 26, 2019 (last R + 13 data collection). The second campaign was performed between September 2, 2019 (first day of BDC 15 days before bed rest) and December 4, 2020 (last data collection 13 days after bed rest), and between September 25 (first day of BDC 14 days before bed rest) and December 23, 2019 (last data collection 13 days after bed rest). Altogether, the subjects each underwent a 15-day BDC phase, a 60-day intervention period, and a 14-day recovery phase. Dietary intakes were standardized based on the same requirements as the VaPER study. As with the VaPER study, all activities were performed in HDT during the bed rest phase, and subjects were instructed not to move their legs besides the physiotherapy and stretching they received to ease excess stiffness or pressure in targeted muscle groups.

### Biological samples

Fasting blood samples and finger stick samples were collected 3 days before bed rest and again on the last day of bed rest. 24-h urine samples were collected 3 days prior to bed rest and again on the day of re-ambulation. DEXA scans were performed 14 days before bed rest and again 11 days after re-ambulation.

Urine and serum calcium were analyzed using an ion-selective electrode (MVZ Lab Quade, Cologne). 25-hydroxyvitamin D was analyzed with ADVIA Centaur Immunoassay (Siemens Healthcare Diagnostic, USA). 1,25-dihydroxyvitamin D was analyzed with chemiluminescent immunoassay (DiaSorin Inc, USA). Parathyroid hormone (PTH) and bone-specific alkaline phosphatase (BSAP) were analyzed with an immune radiometric assay (Immunotech, Czech Republic). Osteocalcin was analyzed with an enzyme-linked immunosorbent assay (ELISA) kit (Techomedical, USA). Type 1 procollagen (P1NP) was analyzed with radioimmunoassay (RIA) (Orion Diagnostica, Finland). Alkaline phosphatase, sodium, magnesium, urea nitrogen, potassium, phosphorus, and chloride were analyzed using a clinical analyzer (Atellica CH Analyzer, Siemens Healthcare Diagnostics Inc, Tarrytown, NY).

Collagen crosslinks N-telopeptide (NTX) and C-telopeptide (CTX) were analyzed in urine samples by ELISA technique (Techomedical, USA and Nordic Bioscience, Denmark, respectively). Deoxypyridinoline (DPD) and pyridinoline (PYD) were analyzed with high performance liquid chromatography (MVZ Lab Quade, Cologne). Crosslink data are presented as both excretion per day and excretion normalized to creatinine. Although 24-h excretion data provides a more reliable reflection than spot samples^55^, in this study, the normalized data help account for the range of body sizes given the mix of men and women in the 2 studies, and the higher proportion of men in the control group.

Three days before bed rest and on the last day of bed rest prior to reambulation, 2 arterialized capillary blood samples obtained from the index or middle finger were collected into heparinized glass capillary tubes and immediately analyzed for PO_2_, PCO_2_ and pH (ABL 800 FLEX; Radiometer, Brønshøj, Denmark).

All biochemistry analyses were performed in a Clinical Laboratory Improvement Amendment (CLIA) certified laboratory.

### Bone densitometry

Bone densitometry was performed using a whole-body densitometer (GE Lunar Prodigy, enCORE software version 16) as previously described^53^. Briefly, single scans were performed for the following sites: whole body, lumbar spine, and hip. Due to other constraints of the studies, the left hip was scanned for VaPER and right hip was scanned for the AGBRESA study.

### Dietary intake

Nutrient intake of both the AGBRESA and VaPER studies were controlled in accordance with the NASA and ESA standardization plan^56^. Total energy requirements for the VaPER study were calculated based on the World Health Organization formula^57^. AGBRESA energy requirements were calculated based on resting metabolic rate measurements. Because energy requirements are lower during bed rest, energy provision was adjusted throughout the studies to maintain the subjects’ body weights within 3% of the weight on the third day of bed rest, at which point fluid redistribution and loss were completed. All subjects consumed standardized meals: a 7-day meal plan before and after bed rest, and a standard 14-day meal plan that was implemented previously for :envihab long-term bed rest studies^43^. Subjects were expected to eat all of their food at each meal and were given an isocaloric diet based on NASA spaceflight nutritional requirements with a balanced intake of macro- and micronutrients and a ratio of 50 to 55% carbohydrate, <35% fat, and 12–15% protein. Subjects consumed a daily standardized level of water based on their weight (50 mL of fluid intake per 1 kg body weight), and they could add lemon juice to the water but were not allowed cocoa, chocolate, tea, alcoholic beverages, herbal drinks, or caffeinated beverages. The results of the vitamin D test during subject screening determined whether a subject received a 2000 IU vitamin D supplement before the HDT phase. Because the efficiency of vitamin D supplementation depends on baseline 25-hydroxyvitamin D and fat and body mass^58^, vitamin D supplementation was initiated 8 weeks prior to the start of bed rest, taking into account body weight and baseline status. The total loading dose used (IU) = 40 × (75 - 25-hydroxyvitamin D (nmol/L) × body weight (kg))^59–62^. Recommended daily doses in this phase ranged from 500–2500 IU vitamin D3/d. All subjects received a daily 1000 IU vitamin D supplement during the HDT phase. Dietary data were recorded with the PRODI Nutrition software (Kluthe, Prodi expert, Stuttgart, Germany).

The Potential Renal Acid Load (PRAL) of the diet was calculated using a formula from Pedone^63^ and Remer^64^: (0.49 × total protein intake) + (0.037 × phosphorus intake) – (0.021 × potassium intake) – (0.026 × magnesium intake) – (0.013 × calcium intake). The absolute PRAL was calculated from the total nutrients consumed, whereas the weight-adjusted PRAL was calculated from the nutrients consumed divided by kg of body mass. Anion gap (AG) was calculated using the equation: AG = [Na^+^] – ([Cl^−^] + [HCO_3_^−^]).

### Statistical analysis

Two-factor repeated measures ANOVA tests were used to determine the group and interactive effect of the treatment (elevated CO_2_ or control) and study phase (before or after bed rest) on the biochemical markers and the bone densitometry in the areas of interest. Post hoc Bonferroni *t*-tests were conducted to determine the difference between groups when the ANOVA yielded significant interactions of *P* < 0.05 (Sigma Plot 12.0, Systat Software Inc., Palo Alto, CA). The biochemical markers were log or 1/x transformed to attain normal distributions. Because serum alkaline phosphatase, serum sodium, serum vitamin K, serum vitamin D, and BSAP could not be normalized, statistics were performed on raw data. The elevated CO_2_ group contained an outlier with values of blood pH greater than 3 standard deviations from the mean. Removing the outlier did not change the statistical significance so the outlier datum was kept. Diet data were not analyzed statistically due to both studies strictly controlling dietary protocols to the same specifications. However, vitamin K status was analyzed and compared between the elevated CO_2_ and control groups because there was a difference in serum vitamin K status between groups.

### Reporting summary

Further information on research design is available in the Nature Research Reporting Summary linked to this article.

## Supplementary information

Reporting Summary

## Data Availability

All data from this paper are available upon request through the NASA Life Science Data Archive.
