# Supplementary material for: Bone metabolism during strict head-down tilt bed rest and exposure to elevated levels of ambient CO2
Source: NPJ Microgravity. 2022 Dec 16;8:57. doi: 10.1038/s41526-022-00245-0 (PMC9758179; doi:10.1038/s41526-022-00245-0)
Supplement: Supplementary file 1 — Reporting Summary [file 41526_2022_245_MOESM1_ESM.pdf]

nature portfolio

Corresponding author(s): Sara R. Zwart

Last updated by author(s): Aug 25, 2022

Reporting Summary

Nature Portfolio wishes to improve the reproducibility of the work that we publish. This form provides structure for consistency and transparency in reporting. For further information on Nature Portfolio policies, see our Editorial Policies and the Editorial Policy Checklist.

Statistics

For all statistical analyses, confirm that the following items are present in the figure legend, table legend, main text, or Methods section.

n/a	Confirmed	
☐	☒	The exact sample size (*n*) for each experimental group/condition, given as a discrete number and unit of measurement
☐	☒	A statement on whether measurements were taken from distinct samples or whether the same sample was measured repeatedly
☐	☒	The statistical test(s) used AND whether they are one- or two-sided<br>*Only common tests should be described solely by name; describe more complex techniques in the Methods section.*
☐	☒	A description of all covariates tested
☐	☒	A description of any assumptions or corrections, such as tests of normality and adjustment for multiple comparisons
☐	☒	A full description of the statistical parameters including central tendency (e.g. means) or other basic estimates (e.g. regression coefficient) AND variation (e.g. standard deviation) or associated estimates of uncertainty (e.g. confidence intervals)
☐	☒	For null hypothesis testing, the test statistic (e.g. *F*, *t*, *r*) with confidence intervals, effect sizes, degrees of freedom and *P* value noted<br>*Give P values as exact values whenever suitable.*
☒	☐	For Bayesian analysis, information on the choice of priors and Markov chain Monte Carlo settings
☒	☐	For hierarchical and complex designs, identification of the appropriate level for tests and full reporting of outcomes
☒	☐	Estimates of effect sizes (e.g. Cohen's *d*, Pearson's *r*), indicating how they were calculated

Our web collection on statistics for biologists contains articles on many of the points above.

Software and code

Policy information about availability of computer code

Data collection	No code was used to collect the data
Data analysis	Data analyses were performed with Sigma Plot 12.0, Systat Software Inc., Palo Alto, CA

For manuscripts utilizing custom algorithms or software that are central to the research but not yet described in published literature, software must be made available to editors and reviewers. We strongly encourage code deposition in a community repository (e.g. GitHub). See the Nature Portfolio guidelines for submitting code & software for further information.

Data

Policy information about availability of data

All manuscripts must include a data availability statement. This statement should provide the following information, where applicable:

- Accession codes, unique identifiers, or web links for publicly available datasets
- A description of any restrictions on data availability
- For clinical datasets or third party data, please ensure that the statement adheres to our policy

All data are available upon request through the NASA Life Sciences Data Archive.

Human research participants

Policy information about studies involving human research participants and Sex and Gender in Research.

Reporting on sex and gender	We did not analyze for sex differences due to the small sample size of females in one of the groups (n=2).
Population characteristics	Inclusion criteria included subjects who were physically and mentally healthy, aged between 24-55 y with a BMI of 19-30 kg/m2. Subjects were required to be non-smokers for at least 6 months, and successfully complete a modified Air Force Class 3 physical. Exclusion criterial included any drug, medication, or alcohol abuse, vegetarian, have migraines, claustrophobia, eye disorders, gastro-esophageal reflux, chronic back complaints, bone fractures in either tibia or radii, muscle or joint disorder, diabetes, or history of chronic bowel disease.
Recruitment	Subjects were recruited by DRL staff
Ethics oversight	NASA and DLR IRBs approved the study protocols

Note that full information on the approval of the study protocol must also be provided in the manuscript.

Field-specific reporting

Please select the one below that is the best fit for your research. If you are not sure, read the appropriate sections before making your selection.

☒ Life sciences ☐ Behavioural & social sciences ☐ Ecological, evolutionary & environmental sciences

For a reference copy of the document with all sections, see nature.com/documents/nr-reporting-summary-flat.pdf

Life sciences study design

All studies must disclose on these points even when the disclosure is negative.

Sample size	The German Space Agency designed the studies and determined sample size based on ocular outcomes. The parameters reported in this paper are a secondary aim of the study and therefore sample size was not calculated based on bone parameters.
Data exclusions	No data exclusions
Replication	All assays were analyzed in triplicate.
Randomization	The data analyses were conducted after the 2 separate studies were complete, therefore subjects were not randomized to each group.
Blinding	Blinding was not relevant to the study. The 2 groups were from studies that were not completed at the same time.

Reporting for specific materials, systems and methods

We require information from authors about some types of materials, experimental systems and methods used in many studies. Here, indicate whether each material, system or method listed is relevant to your study. If you are not sure if a list item applies to your research, read the appropriate section before selecting a response.

Materials & experimental systems

n/a	Involved in the study	
☒	☐	Antibodies
☒	☐	Eukaryotic cell lines
☒	☐	Palaeontology and archaeology
☒	☐	Animals and other organisms
☒	☐	Clinical data
☒	☐	Dual use research of concern

Methods

n/a	Involved in the study	
☒	☐	ChIP-seq
☒	☐	Flow cytometry
☒	☐	MRI-based neuroimaging
